# Integrating pediatric HIV testing and treatment with other child health services in Nigeria

**DOI:** 10.1002/puh2.147

**Published:** 2023-12-20

**Authors:** Muktar Musa Shallangwa, Shuaibu Saidu Musa, Hassan Muhammad Bello, Abdulraz Yahaya, Suraj Abdulkarim Abdullahi, Muhammad Sanusi Ahmad, Mohammed Garba Buwa, Usman Abubakar Haruna, Abdullahi Adamu Malala

**Affiliations:** ^1^ Achieving Health Nigeria Initiative Biu Borno Nigeria; ^2^ Department of Nursing Science Ahmadu Bello University Zaria Nigeria; ^3^ Biu General Hospital Biu Nigeria; ^4^ Institute of Human Virology of Nigeria Katsina Nigeria; ^5^ Department of Planning Research, and Statistics Gombe State Ministry of Health Gombe Nigeria; ^6^ State AIDS & STIs Control Program Gombe State Primary Health Care Development Agency Gombe Nigeria; ^7^ State Specialist Hospital Gombe Nigeria; ^8^ Department of Biomedical Sciences Nazarbayev University School of Medicine Astana Kazakhstan

## INTRODUCTION

Pediatric HIV remains a significant public health challenge, particularly in low‐resource settings. According to UNAIDS, there were an estimated 1.7 million children aged 0–14 years living with HIV in 2021, globally. Despite progress made over the past decade, only 52% of these children had access to treatment in 2021. Interestingly, the number of new HIV infections among children has decreased significantly in recent years, declining by 52% from 320,000 in 2010 to 160,000 in 2021 [1]. However, improving access to testing and treatment for children living with HIV (CLHIV) in Nigeria remains a significant challenge. As a result, the US Centers for Disease Control and Prevention (CDC) initiated the Accelerating Progress in Pediatrics and PMTCT (AP3) program in Nigeria to improve the identification of HIV cases among children and adolescents, as well as the prevention of mother‐to‐child transmission (PMTCT). This will be achieved by promptly identifying and connecting children and adolescents to treatment, as well as boosting pediatric viral suppression rates to 95%, which will help reduce the incidence of illness and fatalities [2], through a range of interventions, including point‐of‐care testing, early infant diagnosis, and integration of HIV services with maternal and child health programs [3]. This can reduce barriers to access and increase uptake of services, by providing a comprehensive package of care that includes prevention, diagnosis, treatment, and support services for both HIV‐ and non‐HIV‐related conditions. This article aims to discuss the opportunities and challenges of integrating pediatric HIV testing and treatment with other child health services in Nigeria.

## EPIDEMIOLOGY OF HIV AND PEDIATRICS HIV CARE

Nigeria has the second‐highest burden of HIV/AIDS in the world, with an estimated 1.8 million people living with the virus at a prevalence of 1.4% (1). The prevalence of HIV/AIDS in Nigeria varies across its different regions, from as high as 3.1% in the South–South–South to as low as 0.6% in the Northwest [4]. In the country, 170,000 children aged 0 to 14 were living with HIV, and 26,000 children were newly infected in 2021 [5]. Despite this burden, only 31% of the children in need of antiretroviral therapy (ART) were receiving it. Although PMTCT efforts reached 34,600 pregnant women, an estimated 100,000 of them needed antiretroviral drugs (ARV) [5]. About 25.17% vertical transmission rate and 7400 new HIV infections were averted through PMTCT, demonstrating a significant progress, but also highlighting the persistent gaps in prevention. In Nigeria, pediatric HIV cases often result from vertical transmissions, which occur when the virus is passed from an HIV‐positive mother to her child during pregnancy, childbirth, or breastfeeding. Vertical transmission remains a significant challenge in the country, underscoring the critical importance of early infant diagnosis (EID) programs in the early identification of infected children before symptoms manifest. Although EID programs are crucial in the early detection of HIV‐infected children, gaps and challenges within the PMTCT program in Nigeria, including inadequate ART coverage for HIV‐infected mothers, late incident infections in pregnant and breastfeeding women, and loss to follow‐up of mother–infant pairs, hinder the optimization of the programs, leading to ongoing transmission and missed case‐finding opportunities, hence underscoring the importance of AP3 program in addressing these gaps [6]. Pediatric HIV care and treatment are primarily provided at hospitals that offer secondary and tertiary‐level specialty services in Nigeria. These services follow national guidelines, which heavily rely on the recommendations provided by the World Health Organization. The guidelines emphasize the importance of initiating ART for all HIV‐positive children, regardless of their clinical stage or CD4+ cell count [7]. This approach ensures that every child with HIV receives the necessary treatment and care, contributing to better health outcomes and an improved quality of life.

## ACCELERATING PROGRESS IN PEDIATRICS AND PMTCT (AP3) PROGRAM

**FIGURE 1:**
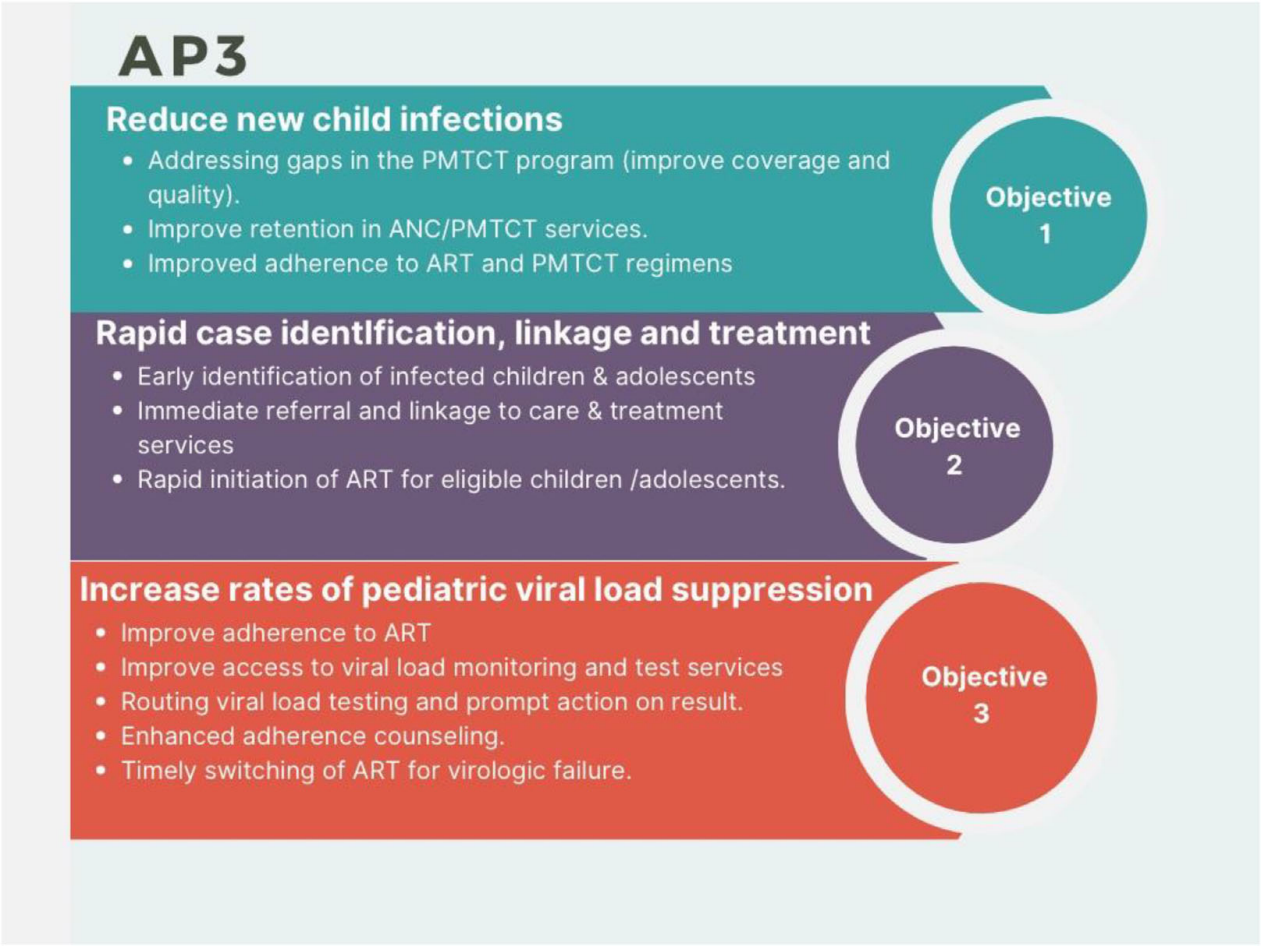
The objectives of Accelerating Progress in Pediatrics and prevention of mother‐to‐child transmission (PMTCT) (AP3) program in Nigeria. AP3: Accelerating progress in pediatrics and PMTCT. ANC: antenatal care. PMTCT: prevention of mother‐to‐child transmission of HIV. ART: antiretroviral therapy.

In order to scale up HIV case identification among pediatric and adolescent populations and PMTCT in Nigeria, the AP3 program was launched in the country with the help of The US Government, through its CDC (2). The program aims to decrease HIV infections in under 10 children by addressing gaps in the PMTCT program, rapidly identifying, and connecting children to treatment, and increasing pediatric viral suppression rates to 95% so as to reduce illness and deaths. It focuses on improving the PMTCT program, enhancing retention in PMTCT services, and improving adherence to ART and PMTCT regimens to reduce new child infections. It also emphasizes early case identification, immediate referral, and rapid initiation of ART for eligible children/adolescents. Lastly, it aims to increase pediatric viral load suppression through improved adherence, enhanced access to viral load monitoring/testing, and timely ART switching for those with virologic failure (see Figure 1).

## RATIONALE OF INTEGRATION

The integration of pediatric HIV testing and treatment with other child health services presents several opportunities to improve access to testing and treatment services and reduce the burden of pediatric HIV. Among the opportunities are the program's potential to increase the uptake of HIV testing and treatment services among children and their caregivers [3]. This can enable caregivers to access these services without having to visit multiple facilities or providers, therefore, reducing the burden of care‐seeking and improve service uptake. By integrating HIV testing into existing maternal and child health services, such as ante‐ and postnatal care, immunization visits, malnutrition clinics, and well‐child clinics, a larger number of pregnant women and infants can be reached, reducing missed opportunities for early diagnosis. This approach enhances coordination among healthcare providers, optimizes resources, and improves overall care for HIV‐positive mothers and infants, leading to better health outcomes and more effective HIV management in Nigeria. It can also help to ensure early initiation of ART among the identified HIV‐positive children, which has been shown to improve health outcomes and reduce transmission of the virus [8].

The integration can also improve the quality of care for HIV‐positive children by ensuring that they receive comprehensive care that includes prevention, diagnosis, treatment, and support services for both HIV‐ and non‐HIV‐related conditions. This can improve the overall health outcomes of HIV‐positive children and reduce the burden of HIV‐related morbidity and mortality in Nigeria. The integration initiative can strengthen health systems by leveraging existing resources and infrastructure meant for providing comprehensive care for HIV‐positive children. It can also reduce stigma and discrimination associated with HIV, by normalizing HIV testing and treatment as part of routine care, therefore, reducing barriers to access and increasing the uptake of services, particularly among vulnerable populations such as adolescents and young children.

## CHALLENGES TO INTEGRATION

Although the integration of pediatric HIV testing and treatment with child health services presents several opportunities, there are several challenges faced by the initiative. Healthcare providers need comprehensive training to deliver integrated services effectively. This includes training on HIV testing and counseling, ART initiation and management, and management of comorbidities and co‐infections. They also need training on how to provide care for vulnerable populations such as adolescents and young children, who may have unique needs related to HIV testing and treatment.

Inadequate supply of laboratory commodities and other supplies such as RTK can be a significant challenge in the successful delivery of integrated services. The consistent availability of these essential commodities is a critical factor that requires utmost attention to overcome these challenges. This requires strong supply chain management systems that can be challenging in low‐resource settings such as Nigeria.

Strengthening health systems could present another challenge to the success of integrated services in Nigeria. This includes ensuring that health facilities are equipped with the necessary infrastructure, equipment, and supplies to provide integrated services. It also includes strengthening health information systems to enable effective monitoring and evaluation of integrated services.

Addressing stigma and discrimination could also be a challenge to improving the uptake of integrated services and reducing the burden of pediatric HIV in Nigeria. CLHIV often face social and psychological challenges due to HIV‐related stigma, which results in delayed diagnosis and treatment initiation [9].

## INTEGRATION STRATEGIES

To improve the implementation of integrated services, several strategies can be employed. Healthcare providers should be provided with comprehensive training on HIV testing and counseling, ART initiation and management, and management of comorbidities and co‐infections. Training should also include strategies to address the stigma and discrimination associated with HIV. Health systems should be strengthened to ensure that health facilities are equipped with the necessary infrastructure, equipment, and supplies to provide integrated services. Health information systems should also be strengthened to enable effective monitoring and evaluation. Task shifting can enable nonspecialist healthcare providers to provide integrated services particularly in settings that are faced with shortages of healthcare workers. Moreover, community engagement and education can help to address HIV‐related stigma and discrimination and increase the uptake of services. Community members should, therefore, be involved in the planning and implementation of integrated services and education and awareness campaigns about HIV to clear misconceptions about the disease and make them aware of the benefits of testing, early diagnosis, and treatment.

## CONCLUSION

Integrating pediatric HIV testing with other child health services can enhance HIV case finding, and HIV/AIDS treatment accessibility and decrease the burden of pediatric HIV in low‐resource settings. Yet, implementing these services faces difficulties, such as healthcare provider training, a steady supply of laboratory commodities, fragile health system, and discrimination. To improve implementation, solutions include comprehensive healthcare provider training, health system enhancement, task shifting, and community involvement. Investing in these strategies not only addresses the challenges but also strengthens the foundation for effective pediatric HIV testing integration, fostering a healthier future for children in resource‐limited settings.

## AUTHOR CONTRIBUTIONS

Muktar Musa Shallangwa conceived the idea. Muktar Musa Shallangwa collected and analyzed the data and drafted the manuscript. Shuaibu Saidu Musa, Hassan Muhammad Bello, Abdulrazak Yahaya, Suraj Abdulkarim Abdullahi, Muhammad Sanusi Ahmad, Mohammed Garba Buwa, Usman Abubakar Haruna, and Abdullahi Adamu Malala rotated in writing different versions of the drafts. All authors read and approved the final manuscript.

## CONFLICT OF INTEREST STATEMENT

Shuaibu Saidu Musa is a Youth Editorial Board member of Public Health Challenges. Therefore, he was excluded from editorial decision‐making related to the acceptance of this article for publication in the journal.

## FUNDING INFORMATION

This research did not receive any specific grant from funding agencies in the public, commercial, or not‐for‐profit sectors.

## Data Availability

Data sharing is not applicable to this article as no new data were created or analyzed in this study.
